# AAV-mediated overexpression of CPT1B protects from cardiac hypertrophy and heart failure in a murine pressure overload model

**DOI:** 10.1007/s00395-025-01123-y

**Published:** 2025-07-11

**Authors:** Anca Kliesow Remes, Theresa Ruf, Tinatin Zurashvili, Lin Ding, Moritz Meyer-Jens, Dominic M. Schwab, Susanne Hille, Andrea Matzen, Sabine Michalewski, Lucia Kilian, Prithviraj Manohar Vijaya Shetty, Marie-Christin Fuchs, Matthias Eden, Hermann-Josef Gröne, Kleopatra Rapti, Andreas Jungmann, Hendrik Milting, Hugo A. Katus, Lucie Carrier, Derk Frank, Norbert Frey, Oliver J. Müller

**Affiliations:** 1 Department of Internal Medicine V, University of Kiel, Kiel, Germany; 2 German Centre for Cardiovascular Research, Partner Site Hamburg/Kiel/Lübeck, Kiel, Germany; 3 Internal Medicine III, University Hospital Heidelberg, Heidelberg, Germany; 4 German Centre for Cardiovascular Research, Partner Site Heidelberg/Mannheim, Heidelberg, Germany; 5 David Tvildiani Medical University, Tbilisi, Georgia; 6 Department of Internal Medicine III, University of Kiel, Kiel, Germany; 7 Department of Experimental Pharmacology and Toxicology, University Medical Center Hamburg Eppendorf, Hamburg, Germany; 8 Department for Cellular and Molecular Pathology, KFZ Heidelberg, Germany; 9 Erich & Hanna Klessmann-Institut, Ruhr-Universität Bochum, Herz & Diabeteszentrum NRW, Bad Oeynhausen, Germany

**Keywords:** Heart failure, CPT1B, Adeno-associated virus, Metabolic remodeling, Fatty acid metabolism, Cardiac hypertrophy

## Abstract

The transition from cardiac hypertrophy to heart failure is characterized by metabolic changes like downregulation of fatty acid metabolism in favor of increased glucose utilization. Carnitine palmitoyltransferase 1B (CPT1B) catalyzes the rate-limiting step of the carnitine shuttle and is an essential enzyme for fatty acid oxidation. Down-regulation of CPT1B activity has been associated with heart failure in patients and various experimental models, indicating an important role in metabolic remodeling. Therefore, we aimed to investigate whether CPT1B overexpression could play a therapeutic role in heart failure. Gene transfer of CPT1B using adeno-associated virus (AAV) vectors into neonatal rat cardiomyocytes significantly attenuated phenylephrine-induced hypertrophy and resulted in decreased generation of mitochondrial reactive oxygen species. In mice subjected to transverse aortic constriction, AAV-mediated cardiac overexpression of CPT1B attenuated cardiomyocyte hypertrophy, cardiac fibrosis, and systolic dysfunction in vivo. Upregulation of CPT1B expression might therefore represent a promising approach to treat or prevent heart failure.

**Supplementary Information:**

The online version contains supplementary material available at 10.1007/s00395-025-01123-y.

## Introduction

To maintain contractility, the heart must continuously produce large amounts of adenosine triphosphate (ATP). It can flexibly switch between energy substrates to sustain this high energy demand. Looking at the respective substrates, the most significant proportion of ATP production derives from the oxidation of fatty acids, followed by glucose, lactate, ketones, and amino acids. However, oxidative phosphorylation of long-chain fatty acids as the most carbon-efficient substrates is less efficient in ATP production per oxygen consumed than glycolysis. The type of substrates used depends on cardiac workload, substrate availability, and hormonal status [25].

Heart failure is a complex clinical syndrome characterized by inadequate cardiac output to maintain sufficient organ perfusion. It is commonly associated with volume overload, reduced cardiomyocyte contractility [4], and coronary microvascular dysfunction [16]. Development of heart failure is associated with metabolic changes, particularly a decrease in metabolic flexibility that allows switching between different energy substrates, resulting in ATP deficiency and impaired contractility [19, 34]. There is a decrease in mitochondrial oxidation and a compensatory increase in glycolysis, which fails to compensate for the energy deficit and only slightly increases ATP production [7, 26, 34]. In addition, these metabolic changes contribute to structural remodeling by activating metabolic signaling pathways [10, 39]. Impaired mitochondrial function is due to various causes, including reactive oxygen species (ROS) production, dysregulation of calcium homeostasis, mitophagy, autophagic cell death of cardiomyocytes, and transcriptional and post-translational changes in mitochondrial proteins [15, 55]. The metabolic changes are complex and depend on the severity and pathogenesis of heart failure and comorbidities. For example, oxidation of fatty acids is decreased in heart failure due to hypertension or ischemia but may be increased in heart failure associated with obesity or diabetes [25].

Metabolic and transcriptional data from cardiac tissue of mice subjected to transverse aortic constriction (TAC) revealed that decreased carnitine shuttling and transportation precedes mitochondrial dysfunction associated with a decreased expression of the enzymes involved like carnitine palmitoyltransferase 1B (CPT1B) [30]. A downregulation of CPT1B was observed in other animal models of heart failure [21, 35]. CPT1 is localized in the mitochondrial outer membrane and catalyzes the rate-limiting step of fatty acid oxidation as it regulates the uptake of long-chain acyl-CoA into mitochondria. The muscle isoform CPT1B is one of three isoforms (CPT1A, B, C) and is the predominant form expressed in the myocardium (98% of CPT1 activity) [48]. CPT1B has been investigated as a potential therapeutic target in animal models and clinical studies with mixed results. Inhibiting CPT1B in heart failure has been shown to improve heart function: CPT1 inhibitors such as etomoxir, perhexiline, and oxfenicine have shown cardioprotective effects in patients in small clinical studies [20, 42] and animal models [23, 45, 46]; specific effects and non-specific off-target effects of CPT1 inhibitors cannot be clearly distinguished. In contrast to those findings, decreased expression of CPT1B, mediated by a cardiospecific prohibitin-2 knock-out, resulted in myocardial accumulation of lipid droplets, reduced mitochondrial maximal respiration, and heart failure, which could be compensated by adenovirus-mediated overexpression of CPT1B [52]. Heterozygous CPT1B knock-out mice subjected to TAC showed lower contractility, mitochondrial abnormalities, myocardial lipid accumulation, and increased apoptosis [14]. Homozygous CPT1B deficiency is lethal [18].

Due to these conflicting results, this study aimed to elucidate the effect of CPT1B overexpression in developing cardiac hypertrophy and heart failure in mice.

## Materials and methods

### Vector cloning, production, and quantification

cDNA encoding full-length CPT1B was amplified using the forward (5’-TCAGTCTCTAGAGCCACCTGGCGGAAGCACACC-3’) and reverse primers (5’-TCAGTCTCTAGACCTCAGCTGTCTGTCTTGG-3’) and inserted via the XbaI restriction site into a cytomegalovirus (CMV)-enhanced 1.5 kb myosin light chain (MLC) promoter [31, 49]. A single-stranded luciferase reporter gene from the same vector genome background was used for controls. AAV6 and AAV9 vectors were produced either with the CPT1B or luciferase genomic plasmid, purified using iodixanol step gradient ultracentrifugation, and titrated by qPCR of the vector genomes as previously described [49].

### NRVCM isolation and treatments

Neonatal rat ventricular cardiomyocytes (NRVCMs) were isolated as previously published. Briefly, left ventricles from 1- or 2-day-old Wistar rats (Charles River) were harvested and sectioned in pH 7.4 buffer containing 120 mmol/L NaCl, 20 mmol/L Hepes, 8 mmol/L NaH_2_PO_4_, 6 mmol/L glucose, 5 mmol/L KCl, and 0.8 mmol/L MgSO_4_. Separation of individual cardiomyocytes from sectioned tissue mass was achieved by enzymatic digestion with 0.6 mg/ml pancreatin (Sigma-Aldrich) at 37 ℃ and 0.5 mg/ml collagenase type II (Worthington Biochemical Corporation). Cardiomyocytes were separated from fibroblasts using a Percoll gradient centrifugation step (GE Healthcare). Thereafter, cardiomyocytes were cultured in DMEM medium containing 10% fetal calf serum (FCS), 2 mmol/L penicillin/streptomycin, and L-glutamine (PAA Laboratories). NRVCMs were transduced with an AAV serotype 6 expressing CPT1B (AAV6-CPT1B) or Luciferase (AAV6-Luc). A multiplicity of infection (MOI) of 1 × 10^5^ was used. Phenylephrine (PE, 100 nmol/L), endothelin-1 (ET-1, 200 nmol/L) or isoprenaline (100 mmol/L) in serum-free medium were added 3 days after transduction. Palmitate-BSA or BSA (Agilent) was added to transduced cells to a concentration of 500 μmol/L. Hypoxia was achieved by incubating cells under 1% O_2_ conditions for 48 h in glucose-free medium.

### Culture of human induced pluripotent stem cells (hiPSCs) and hiPSC-derived cardiomyocytes (hiPSC-CMs)

The control hiPSC-line mTagRFPT-TUBA1B (AICS-0031-035, Coriell Institute, Camden, NJ, US) was used for this study. HiPSCs were seeded at a density of 6.5 × 10^4^ hiPSC/cm^2^ in in-house medium (DMEM F-12, 21331046, Gibco; L-glutamine 2 mmol/L, 25030081, Gibco; transferrin 5 µg/mL, T8158. Sigma; selenium 5 ng/mL, S5261, Sigma; human serum albumin 0.1%, 05-720-1B, neoFroxx; lipid mixture 1X, L5146, Sigma; insulin 5 µg/mL, I9278, Sigma; dorsomorphin 50 nM, S7306, Selleckchem; activin A 2.5 ng/ml, 78132.2, Stemcell Technologies; TGFβ1 0.5 ng/ml, 100-21C, Peprotech; bFGF 30 ng/ml, 100-18B, Peprotech) on Geltrex (A14133-02, Gibco)-coated culture ware and cultivated under hypoxic conditions. Medium was exchanged daily and hiPSCs were split at 90% confluency. For this step, the regular culture medium was supplemented with 10 µmol/L Y-27632 (orb154626, Biorbyt). HiPSCs were differentiated to hiPSC-CMs using a monolayer protocol (adapted from [29]. In short, cells were seeded onto Matrigel (354234, Corning) coated 6-well plates on day 1 at 5.2 × 10^4^ hiPSC/cm^2^. The next day, they were fed in the morning with culture medium as described above. Between 7 and 9 PM, medium was exchanged for stage 0 medium (StemPro-34 SFM, StemPro Supplement 2.6%, 10639011, Gibco; BMP4 1 ng/mL, 314-BP, R&D Systems; L-glutamine 2 mmol/L, Gibco; Matrigel, 354234, Corning). After 14 h, stage 0 medium was replaced by stage 1 medium (StemPro-34 SFM, StemPro supplement 2.6%; BMP4 10 ng/mL; L-glutamine 2 mmol/L; activin A 8 ng/mL, Stemcell Technologies). Stages 2.1 (RPMI1640, Gibco; B27 2%, in-house; KYO2111 10 µmol/L, 4731, Tocris; XAV939 10 µmol/L, 3748, Tocris) and 2.2 (stage 2.1 with insulin 3.21 µg/mL, Sigma) followed after 48 h of incubation with the previous medium each. Another 2 days after stage 2.2, cells were fed (RPMI1640, B27 2%, insulin 3.21 µg/mL) every other day until they started to beat homogenously. Cells were dissociated as described before [29] and seeded onto SeaHorse specific 96-well plates coated with Geltrex in complete culture medium (DMEM, D5546, Sigma; 10% FCS, 26140079, Invitrogen; 1% Pen-Strep, 15140122, Gibco; 10 µg/mL Insulin) at a density of 4 × 10^4^ cells/well. After the cells had attached to the culture dish, medium was changed to maturation medium (DMEM w/o glucose, 11966025, Gibco; glucose 3 mmol/L, G7021, Sigma; L-lactate 10 mmol/L, 71718, Sigma; vitamin B12 5 µg/mL, V6629, Sigma; biotin 0.82 µmol/L, B4639, Sigma; creatine monohydrate 5 mmol/L, C3630, Sigma; taurine 2 mmol/L, T0625, Sigma; L-carnitine 2 mmol/L, C0283, Sigma; ascorbic acid 0.5 mM, A8960, Sigma; NEAA 1X, 11140, Thermo Fisher Scientific; albumax I 0.5%, 11020021, Thermo Fisher Scientific; B27 1X, in-house; Knockout serum replacement 1X, 10828028, Thermo Fisher Scientific; penicillin–streptomycin 1%, insulin 10 µg/mL) with medium changes every 5 days for a total of 14 days. Afterwards, hiPSC-CMs were transduced and treated with the indicated viruses (AAV-CPT1B, AAV-Luc).

### BODIPY staining

To determine the degree of fatty acid accumulation in NRVCMs, cells were subjected to BODIPY staining as previously described [6]. In brief, cells were cultured on glass coverslips and incubated with 2 μmol/L BODIPY (Thermo Fischer Scientific) for 30 min at 37 °C. Afterwards, cells were fixed by incubation with 4% PFA, and nuclei were stained with DAPI. Imaging was performed using confocal microscopy (LSM 800, Zeiss). Images were analyzed using ImageJ (FIJI, version 1.54p).

### Animal experiments

Animal experiments were carried out under the guidelines from directive 2010/63/EU of the European Parliament on the protection of animals used for scientific purposes with the approval of the Karlsruhe Regional Council (35-9185.81/G-122/12). The animals were housed under standard conditions (Interfaculty Biomedical Research Facility (IBF), Heidelberg, Germany) with a 12-h light/12-h night cycle. Water and food were offered ad libitum. Ten-week-old C57BL/6N mice were injected with the AAV9 vectors intravenously via the tail vein. We injected 3 × 10^12^ vg AAV9-CMV-MLC1500-Luc for the control group or 3 × 10^12^ vg AAV9-CMV-MLC1500-CPT1B for the intervention group; sham mice were not injected. Transverse aortic constriction was performed 2 weeks later, as previously described [40] using a 27-gauge needle for stenosis induction. Successful ligation was confirmed by measuring the right carotid/left carotid flow velocity ratio. Cardiac function was determined at baseline (before TAC surgery) and every 2 weeks by echocardiography using the VisualSonics Vevo 2100 imaging system and the 40 Hz MS-550D Microscan transducer. An investigator who was blinded to treatment performed measurements and data analysis. Long-axis and short-axis M-mode cine loops were recorded. Ejection fraction, fractional shortening, and left ventricular end-diastolic diameter (LVEDD) were determined using VisualSonics software. The animals were killed 6 weeks after the operation (study design see Fig. 4a).

### Human samples

Myocardium samples were obtained from patients diagnosed with hypertrophic cardiomyopathy (HCM), dilated cardiomyopathy (DCM), and ischemic cardiomyopathy (ICM). Non-failing (NF) samples derived from rejected donor hearts were used as controls. The study was approved by the responsible Medical Ethical Committee (number D462/15 and D506/22). 10 biopsies were used in each patient cohort, while 3 non-failing samples were analyzed in the control group. All patients were males, with a mean age of 52.8 years (DCM patients), 53.2 years (ICM patients), 52.6 years (HCM patients), and 51.6 years (controls).

### RNA isolation, cDNA synthesis and relative quantification qPCR

RNA was extracted and purified from NRVCM using an RNA extraction kit (Zymo Research) according to the manufacturer’s instructions. Reverse transcriptase (BioLab) and oligo(dT) primers were used to reverse transcribe 1 μg of RNA into cDNA according to the manufacturer’s specifications. RT-qPCR was performed with SYBR Green Supermix (Thermo Fisher Scientific) and a CFX96 Touch Real-Time PCR Detection System (Bio-Rad). The following primer sequences were used: RPL32 forward 5´-TCGGCCTCTGGTGAAG-3´, RPL32 reverse 5´-AGGACACATTGTGAGCAATC-3´; CPT1B forward 5-CCCATGTGCTCCTAC-3´, CPT1B reverse 5´-CGACCATTCTCTGGA-3´; NPPA forward 5´-ACCTGCTAGACCACCTGGAGGAG-3´, NPPA reverse 5´-CCTTGGCTGTTATCTTCGGTACCGG-3´; NPPB forward 5´-TGATTCTGCTCCTGCTTTTC-3´, NPPB reverse 5´-GTGGATTGTTCTGGAGACTG-3´; β-MHC forward 5´-TGCAAAGGCTCCAGGTCTGAGGGC-3´, β-MHC reverse: 5´-GCCAACACCAACCTGTCCAAGTTC-3´. Three technical replicates were used for each reaction. RPL32 served as the housekeeping gene, and values were normalized to the control samples.

Left ventricular tissue was processed using the TissueLyser (Qiagen, Hilden, Germany). Total RNA was extracted and purified from heart tissue using the EURx GeneMATRIX Universal RNA Purification Kit (Roboklon, Berlin, Germany) according to the manufacturer’s instructions. First-strand synthesis of cDNA was performed using the SuperScript® III First-Strand Synthesis System for RT-PCR (Invitrogen/Life Technologies) and dNTPs and OligodT primers (Promega, Mannheim, Germany) using the manufacturer’s protocol and starting from equal amounts of RNA for each sample. qRT-PCR was performed with SYBR Green Supermix (Thermo Fisher Scientific) and a CFX96 Touch Real-Time PCR Detection System (Bio-Rad). The following primer sequences were used: GAPDH forward 5´-ATGTTCCAGTATGACTCCACTCACG-3´, GAPDH reverse 5´-GAAGACACCAGTAGACTCCACGACA-3´; CPT1B forward 5´-CCCATGTGCTCCTACCAGAT-3´, CPT1B reverse 5´-CGAGGATTCTCTGGAACTGC-3´; NPPA forward 5´-ACCTGCTAGACCACCTGGAGGAG-3´, NPPA reverse 5´-CCTTGGCTGTTATCTTCGGTACCGG-3´; NPPB forward 5´-GGTCTTCCTACAACAACTTCAG-3´, NPPB reverse 5´-GGTCTTCCTACAACAACTTCAG-3´; β-MHC forward 5´-GCCAACACCAACCTGTCCAAGTTC-3´, β-MHC reverse 5´-TGCAAAGGCTCCAGGTCTGAGGGC-3´; Col1A1 forward 5´-CCCGCCGATGTCGCTATCCA-3´, Col1A1 reverse 5´-CAGCAGGGCCCTTTCCTCCC-3´; Col3A1 forward 5´-TGGCCCAGCTGGTGACAAGG-3´, Col3A1 reverse 5´-CAGCAGGGCCCTTTCCTCCC-3´. Two technical replicates were used for each reaction. GAPDH served as the housekeeping gene, and values were normalized to the control samples.

### Immunofluorescence analysis and determination of cell size

NRVCMs were cultured on coverslips and fixed with 4% paraformaldehyde in PBS for 20 min at room temperature. Next, non-specific binding sites of antibodies were blocked with a PBS buffer containing 5% BSA and 0.1% Triton X-100 for 1 h at room temperature. After that, the cells were incubated overnight with the primary antibodies (CPT1B, α-actinin, 1:200) at 4 ºC. After washing with PBS, the cells were incubated with an Alexa-594- or Alexa-488-labeled secondary antibody (Thermo Fisher Scientific, Darmstadt, Germany, 1:400) for 1 h. Cell nuclei were visualized by staining with 1 µg/ml 4',6-diamidino-2-phenylindole (DAPI). The coverslips were then mounted on microscope slides with FluorSave reagent. Approximately 100 cells were randomly selected for each group for cell size measurement and photographed using a fluorescence microscope (BZ-X800, Keyence). Image J (1.51p, National Institute of Health, US) was used for the measurement of fluorescence intensity and cell size.

Deparaffinized sections were incubated with a sodium citrate buffer for 10 min at 95 °C and treated with a buffer containing 2.5% BSA and 0.1% Triton X-100 for 1 h. After that, the sections were incubated overnight with the primary antibody (CPT1B, LS-C490167, LS-Bio, 1:500) at 4 °C in a humidified atmosphere. After washing with PBS, the sections were incubated with the Alexa 546-labeled secondary antibody (Thermo Fisher Scientific, Darmstadt, Germany, 1:400) for 1 h. Cell nuclei were visualized by staining with 1 µg/ml 4',6-diamidino-2-phenylindole (DAPI). Wheat Germ Agglutinin (WGA, Thermo Fischer Scientific, Bremen, Germany) was used for cell membrane staining. A confocal microscope (Zeiss LSM 800, Oberkochen, Germany) was used to acquire images. Image J was used for the measurement of fluorescence intensity and cell size.

### Quantification of fibrosis

Hearts were fixed in 4% PFA overnight at 4 °C and embedded in paraffin. Sections were subjected to Masson’s Trichrome Staining (Sigma-Aldrich, Munich, Germany) according to the manufacturer’s instructions to visualize extracellular matrix deposition. Images were acquired in random areas of the left ventricle using a bright-field microscope (Leica DM500, Leica Microsystems, Mannheim, Germany). The percentage of the blue area over the total area of the section was measured using ImageJ.

### Measurement of mitochondrial function

The membrane-potential-dependent dye tetramethylrhodamine ethyl ester (TMRE, Molecular Probes) was used to monitor mitochondrial membrane potential (ΔΨ_m_). Isolated cardiomyocytes were loaded with TMRE at a final concentration of 100 nmol/L by incubation at 37 ºC for 30 min and then kept in phenol red-free DMEM medium (Thermo Fischer Scientific) until visualization. Images were acquired using confocal microscopy (Zeiss, LSM800) and analyzed using ImageJ.

### Measurement of mitochondrial ROS

Mitochondrial superoxide production was monitored by incubating the cells with 5 μmol/L MitoSOX Red Mitochondrial Superoxide Indicator (Invitrogen) in serum-free DMEM at 37 ºC for 30 min followed by washout. MitoSOX fluorescence was measured. The visualization was performed using confocal microscopy with excitation at 576 nm and measurement of emitted light at 585 nm. Quantitative analysis was performed with ImageJ.

### Seahorse analysis

Glycolysis stress test and fatty acid oxidation assay were performed according to manufacturer’s instructions, using the XF96 Analyser (Agilent). In brief, 50,000 NRVCMs were plated on collagen-coated 96-well Seahorse plates and transduced with AAV6-CPT1B or AAV6-Luc at a MOI of 10^5^ virus particles/cell. Seahorse assay was performed 3 days after transduction, using at least 12 technical replicates per experimental group.

#### Glycolysis stress test

Culture medium was replaced with low buffered, phenol red-free DMEM (Agilent), supplemented with 2 mmol/L-glutamine, and further cells were incubated under CO2-free conditions at 37 °C prior to running the assay. The compounds and corresponding concentrations used during the assay are as follows: glucose (10 mmol/L), oligomycin (1 μmol/L), and 2-DG (50 mmol/L). Oxygen consumption rate (OCAR) and extracellular acidification rate (ECAR) were constantly measured during the procedure. Data analysis was performed using Wave software (Agilent).

#### Palmitate oxidation

Culture medium was changed to substrate-limited medium (DMEM Corning, with 0.5 mmol/L glucose, 1 mmol/L-glutamine, 0.5 mmol/L-carnitine) and cells were cultured overnight. One hour before the assay start, medium was exchanged for substrate-limited FAO medium (NaCl 111 mmol/L, KCl 4.7 mmol/L, CaCl_2_ 1.25 mmol/L, MgSO 2 mmol/L, NaH_2_PO_4_ 1.2 mmol/L, 2.5 mmol/L glucose, 0.5 mmol/L-carnitine, and 5 mmol/L HEPES). After 45 min, etomoxir (Merck) was added to a final concentration of 40 μmol/L. Further, palmitate-BSA or BSA was added (500 μmol/L).

### Cell viability assays

NRVCMs were seeded in 24-well plates at a density of 150,000 cells/well and transduced either with AAV6-CPT1B or AAV6-Luc. Three days after transduction, cells were subjected to hypoxia (1% O_2_) for 48 h in glucose-free DMEM (Thermo Fischer Scientific). For total metabolic activity, 3-(4,5-dimethylthiazol-2-yl)-2,5-diphenyltetrazolium bromide (MTT) assay was used according to the manufacturer’s instructions (Merck). In brief, cells were treated with 30 μL MTT reagent, incubated for 4 h at 37 °C, followed by the addition of 400 μL solubilization buffer. Afterwards, absorbance was measured at 590 nm (with reference at 690 nm). Lactate dehydrogenase (LDH) release was measured using a specific kit (CyQUANT, Thermo Fischer Scientific) according to the instructions provided.

### Statistics

The evaluation of the statistical data and the generation of graphs was carried out with the software GraphPad Prism 5 (San Diego, California, USA). Differences between 3 or more different groups were assessed using one-way ANOVA, followed by Newman–Keuls multiple comparisons test for specific pairs of groups. Mann–Whitney *U* test was used to compare two groups. A p value < 0.05 was considered significant. Data are presented as mean ± standard deviation of individual experiments.

## Results

### CPT1B expression is decreased in pro-hypertrophic conditions

**Fig. 1:**
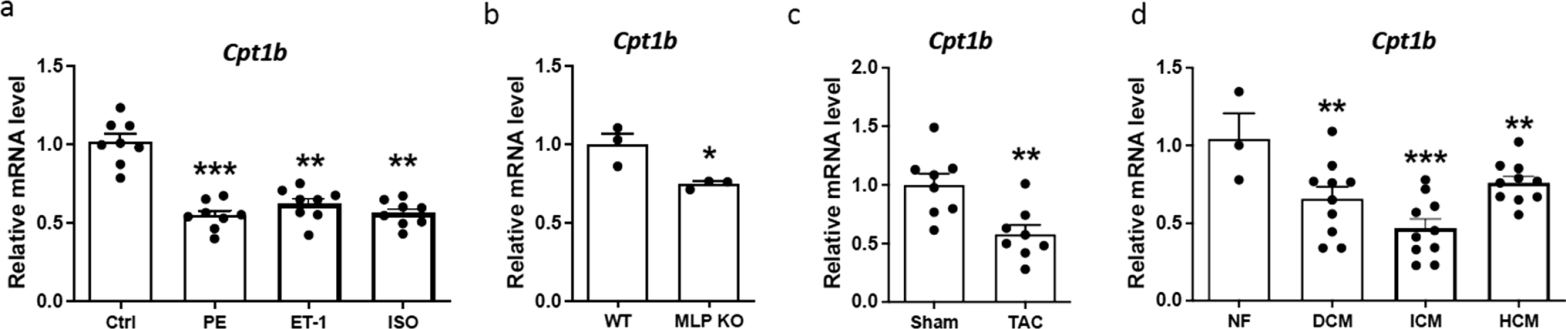
CPT1B mRNA level is lower in various models of heart insufficiency. **a** Statistical quantification of CPT1B mRNA levels in NRVCMs after treatment with phenylephrine (PE), endothelin-1 (ET-1) or isoprenaline. Values were normalized to untreated cardiomyocytes as a control. *n* = 6 **b** Statistical quantification of CPT1B mRNA levels in MLP knock-out (MLP-KO) compared to, normalized to wild-type littermates, n = 3. **c** Statistical quantification of CPT1B mRNA levels in mice after transverse aortic constriction (TAC) vs. sham-operated mice, normalized to sham mice. *n* = 8. **d** Statistical quantification of CPT1B mRNA levels in human myocardial biopsies from non-failing controls and patients with hypertrophic cardiomyopathy (HCM), dilated cardiomyopathy (DCM) and idiopathic cardiomyopathy (ICM). GAPDH (**a**-**c**) and RPL32 (**d**) were used as a housekeeping gene, *n* = 3 for control (non-failing, NF) and *n* = 10 for patient samples. (*p < 0.05, **p<0.01, ***p < 0.001, quantitative data are presented as means ± SD)

First, we aimed to investigate the differential expression of CPT1B in various models of heart failure compared to healthy controls. In vitro, we used PE, ET-1, and isoprenaline (ISO) to induce hypertrophy in NRVCM, resulting in a significant downregulation of CPT1B (Fig. 1a). To study two in vivo heart failure models, we compared muscle lim protein (MLP) knock-out mice and TAC mice with corresponding controls (healthy littermates and sham-operated mice, respectively). As shown in Fig. 1b, c, CPT1B mRNA levels were significantly lower in both hypertrophic phenotype groups. Finally, we examined myocardial biopsies from patients with hypertrophic cardiomyopathy (HCM), dilated cardiomyopathy (DCM), and ischemic cardiomyopathy (ICM), which all exhibited lower CPT1B mRNA levels than in healthy hearts (Fig. 1d).

### AAV6-mediated overexpression of CPT1B ameliorates PE-induced cardiomyocyte hypertrophy in NRVCMs

**Fig. 2:**
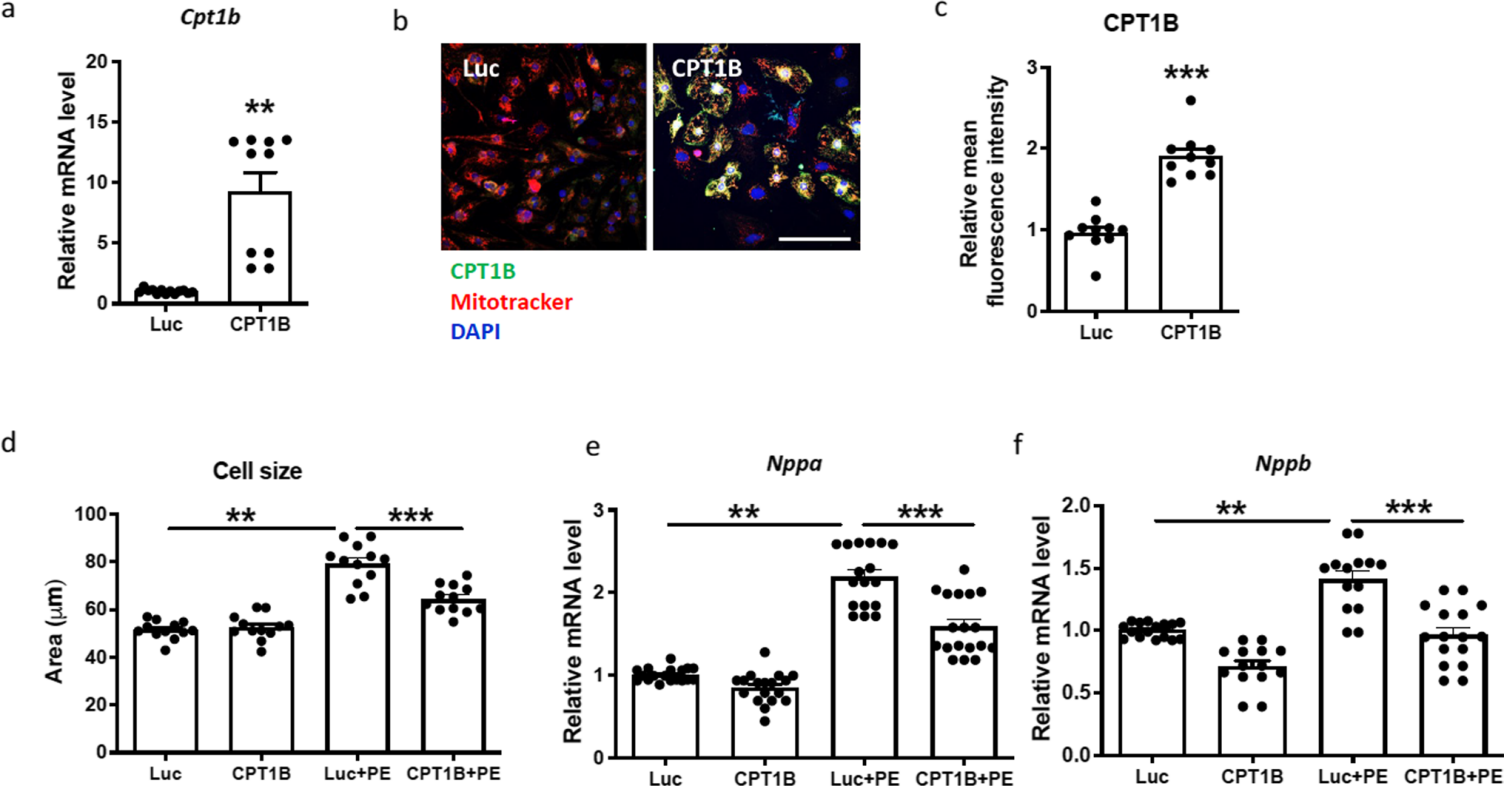
AAV6-mediated overexpression of CPT1B ameliorates PE-induced cardiomyocyte hypertrophy in NRVCMs. **a** Statistical quantification of CPT1B mRNA levels in NRVCMs after transduction with AAV6. **b** Representative images showing CPT1B immunohistochemistry (green). Mitochondria were labeled with the mitotracker dye (red), and nuclei were stained with DAPI (blue); scale bar = 25 µm. **c** Statistical quantification of mean green fluorescence intensity in transduced cells. Sixteen images were analyzed/condition.** d** Statistical quantification of cardiomyocyte cross-sectional area, measured following staining with α-actinin 2 as a cytoskeleton protein. **e, f** Quantification of fetal gene program in NRVCMs in the depicted treatment groups. (**p < 0.01, ***p < 0.001, one-way ANOVA, quantitative data are presented as means ± SD, *n* = 4)

Next, we aimed to achieve overexpression of CPT1B in NRVCMs by AAV6 transduction and to assess its role in pro-hypertrophic conditions. As shown in Fig. 2a, the CPT1B mRNA level was significantly higher in NRVCMs transduced with AAV6-CPT1B than in AAV6-Luc-transduced cells. In addition, the mitochondrial localization of the overexpressed protein was confirmed by immunocytochemistry analysis (Fig. 2b, c).

Furthermore, we examined the functional effect of CPT1B overexpression on the PE-mediated hypertrophic response in cardiomyocytes. For this purpose, we determined whether overexpression of CPT1B affects the NRVCM surface, which was analyzed by immunostaining with α-actinin 2. As shown in Fig. 2d, PE treatment induced a marked increase in cardiomyocyte size compared to untreated cells in the AAV6-Luc treatment group. On the other hand, CPT1B-overexpressing cells presented a significantly reduced cell size even with the pro-hypertrophic stimulus.

In addition, we determined the mRNA levels of the well-characterized markers *Nppa* and *Nppb*. The mRNA levels of the natriuretic peptides have been shown to be reactivated during the development of cardiac hypertrophy and hence serve as markers for the disease both in vitro and in vivo [12]*.* As expected, PE treatment induced a significant accumulation of both *Nppa* and *Nppb* mRNA levels in AAV6-Luc-transduced control cells but not in AAV6-CPT1B-transduced NRVCMs (Fig. 2e, f).

To determine whether CPT1B overexpression is protective in other models of cardiac dysfunction, we subjected NRVCMs to palmitic acid treatment and hypoxia as models for obesity-induced cardiac injury and myocardial infarction, respectively. As demonstrated in Suppl. Figure 1A, B, NRVCMs treated with AAV-CPT1B presented with a significant decrease in palmitate-induced hypertrophy as compared to AAV-Luc-treated cells. In contrast, CPT1B overexpression did not rescue hypoxia-induced cell death, demonstrated by MTT assay and LDH release test (Suppl. Figure 1C, D).

Taken together, these data suggest that CPT1B overexpression attenuates PE- and palmitate-induced hypertrophy in cardiomyocytes in vitro but does not influence cardiomyocyte dysfunction under hypoxic conditions.

### AAV6-mediated overexpression of CPT1B blunts PE-induced mitochondrial dysfunction

**Fig. 3:**
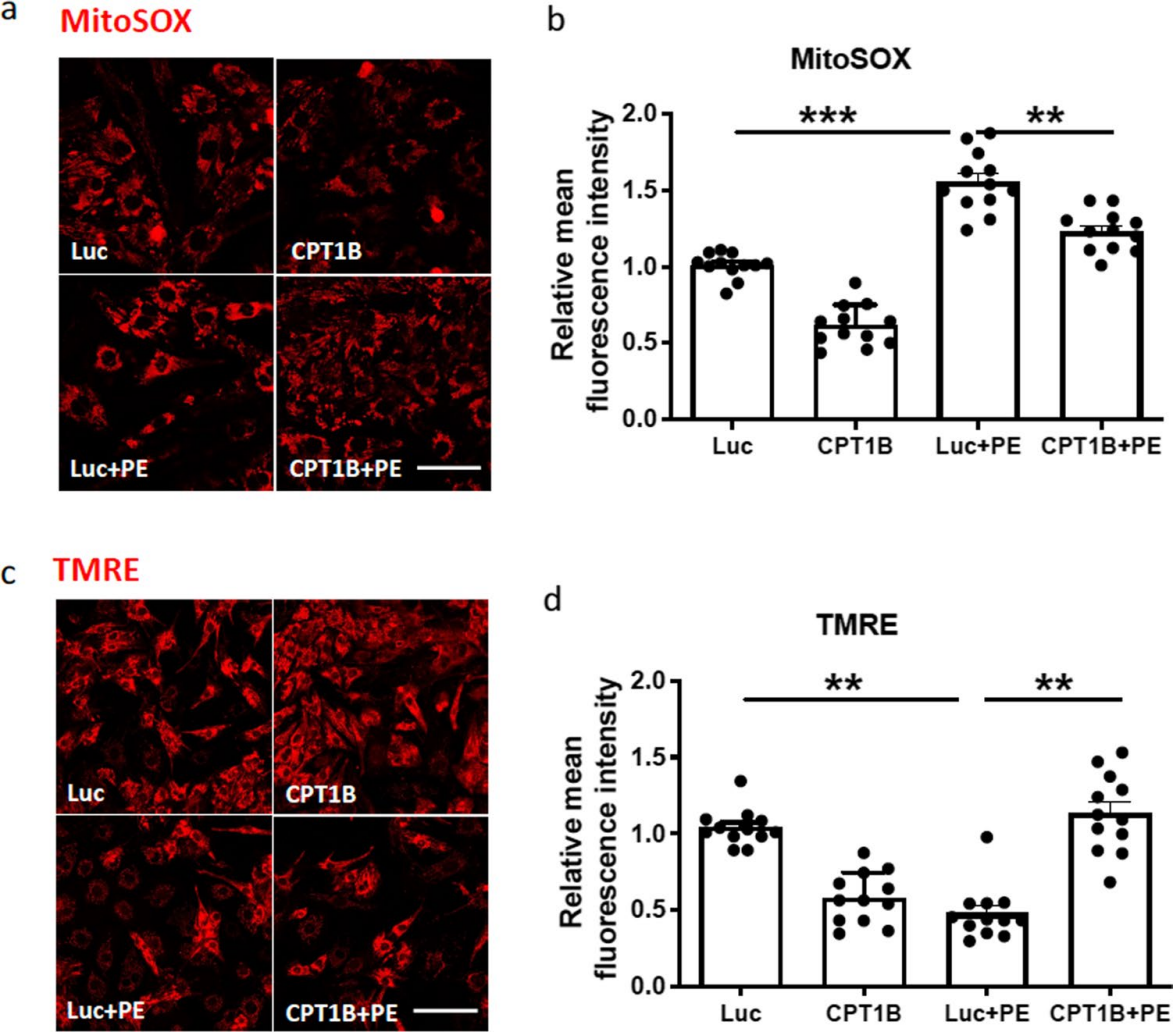
AAV6-mediated overexpression of CPT1B ameliorates PE-induced mitochondrial dysfunction. **a** Illustrative images showing MitoSOX staining (red) of NRVCMs subjected to the depicted treatments. **b** Statistical quantification of relative red fluorescent signal measured by ImageJ. Cells transduced with AAV6-Luc served as controls. **c** Representative confocal images of NRVCMs treated with TMRE as an indicator of mitochondrial membrane potential and **d** quantification of relative mean red fluorescence intensity; scale bar = 25 µm. (**p < 0.01, ***p < 0.001, one-way ANOVA, quantitative data are presented as means ± SD, *n* = 4, 16 images analyzed/condition)

**Fig. 4:**
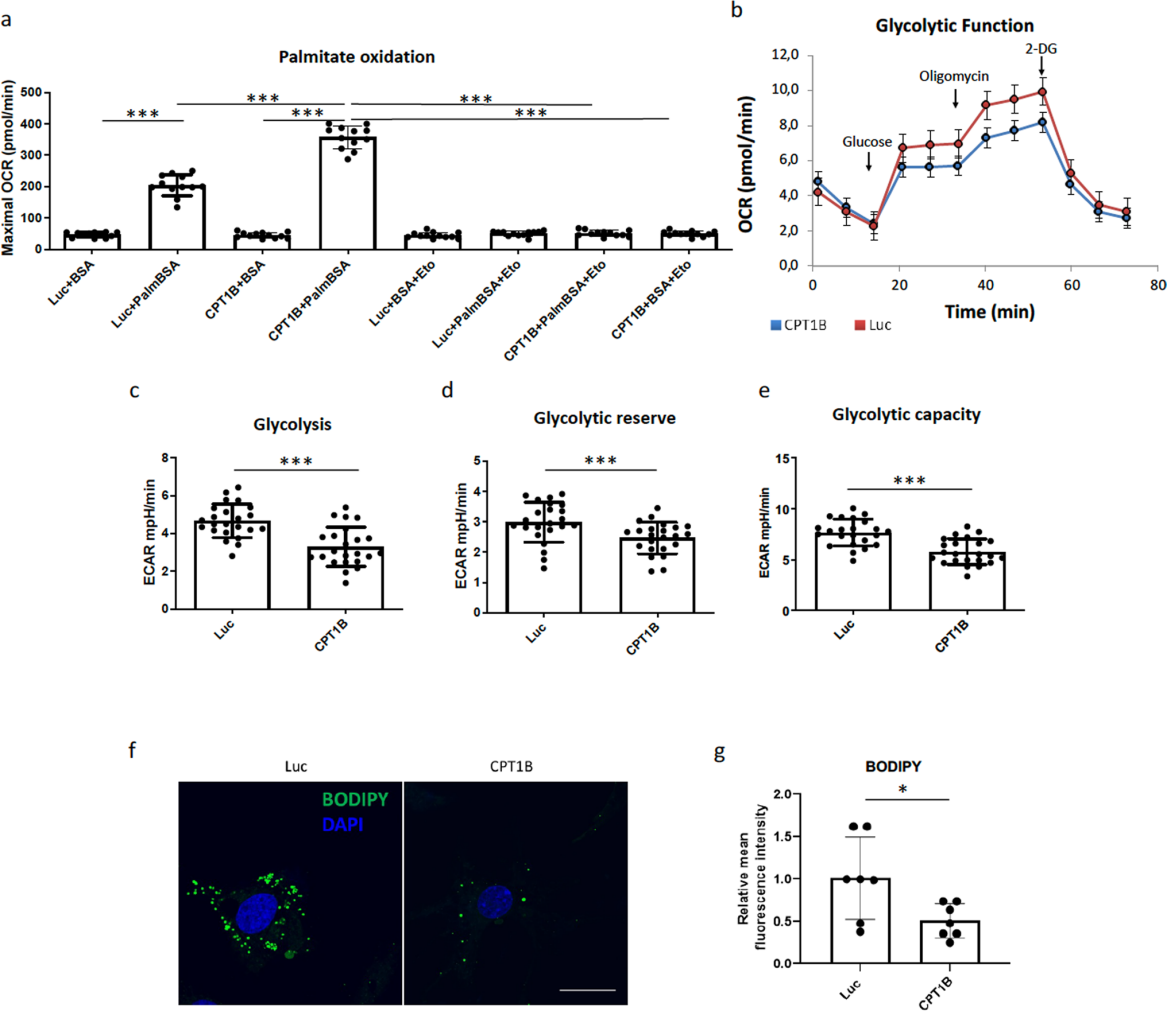
CPT1B overexpression induces palmitate oxidation in NRVCMs. **a** Maximal respiration rate, as a measure of palmitate oxidation in the depicted experimental groups. BSA: bovine serum albumin, PalmBSA: saturated fatty acid complex composed of 6:6 palmitate:BSA, Eto: etomoxir.** b** Representative diagram showing oxygen consumption rate (OCR) acquired during glycolysis stress test assay. **c, d, e** Assessment of glycolysis, glycolytic reserve and glycolytic capacity following CPT1B overexpression in NRVCMs, measured by Seahorse assay. **f** Representative images showing BODIPY staining in NRVCMs subjected to AAV6-CPT1B and AAV6-Luc transduction and **g** statistical quantification of mean fluorescence intensity, as a marker of lipid droplets accumulation in cardiac myocytes (***p < 0.001, one-way ANOVA, quantitative data are presented as means ± SD, *n* = 3)

Mitochondrial dysfunction and increased oxidative stress are established hallmarks of failing hearts [17, 35]. Therefore, we next aimed to investigate whether CPT1B overexpression improves mitochondrial activity under stimulation with PE. As expected, our analyses show a significant increase in mitochondrial ROS, superoxide in particular, after PE stimulation, detected by staining with MitoSOX. AAV-mediated CPT1B overexpression led to the normalization of oxidative stress (Fig. 3 a, b). Cells were further subjected to live cell imaging after incubation with TMRE to assess changes in mitochondrial membrane potential. As shown in Fig. 3c, d, the application of AAV6-CPT1B prior to PE stimulation resulted in a significant increase in mitochondrial membrane potential compared to cells treated with AAV6-Luc. We next aimed to determine whether CPT1B overexpression affects palmitate oxidation and glycolytic function by Seahorse assay. As shown in Fig. 4a-e, a beneficial effect on metabolic function was observed in NRVCM, where CPT1B overexpression resulted in a significant increase in the level of fatty acid oxidation, while the glycolytic capacity of NRVCMs was markedly decreased. In consistency, we could confirm a marked decrease in the accumulation of lipid droplets in CPT1B-overexpressing NRVCMs in comparison to controls (Fig. 4f,g). To confirm the aforementioned results in a suitable human cardiac cell model, we subjected hiPSC-CMs to the Seahorse assay following CPT1B and Luc transduction. As demonstrated in Supplementary Fig. 2, cells transduced with AAV6-CPT1B presented with a significant increase in palmitate oxidation as compared to AAV6-Luc treated cells, confirming that the overexpressed protein is functional in both the rat and human cardiomyocyte models.

### AAV9-mediated overexpression of CPT1B prevents pressure overload-induced heart failure

**Fig. 5:**
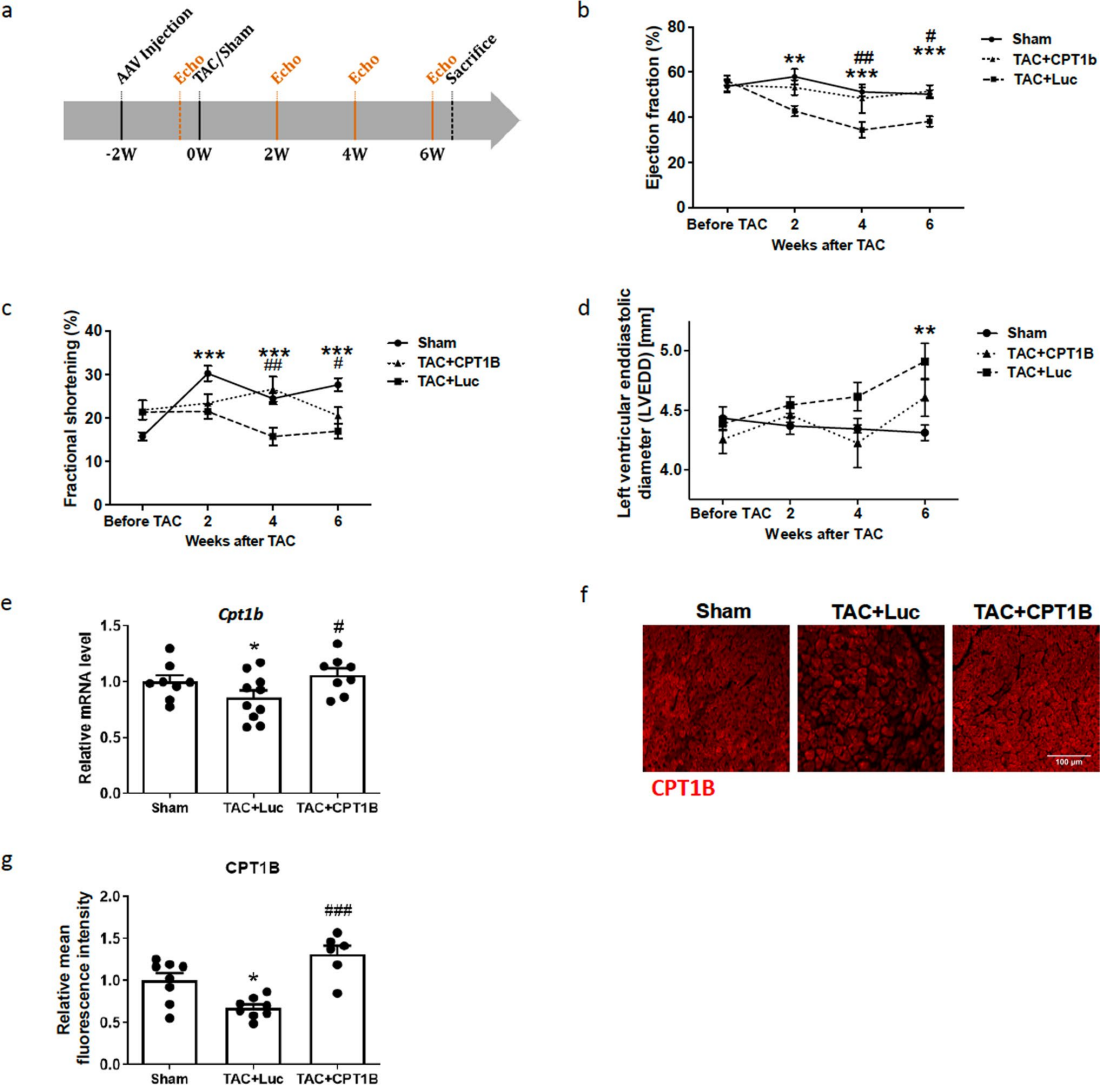
AAV9-mediated overexpression of CPT1B in cardiomyocytes prevents deterioration of cardiac function.** a** Graphical representation of the experimental timeline (w = weeks). **b** Echocardiographic measurement of ejection fraction over the four time points (baseline; 2, 4, and 6 weeks after TAC). **(c)** Echocardiographic measurement of fractional shortening over the four time points.** d** Echocardiographic measurement of the left ventricular end-diastolic diameter over the four time points. **e** Statistical quantification of CPT1B mRNA levels in cardiac tissue of mice subjected to the depicted treatments. Values were normalized to GAPDH, which served as housekeeping gene and expressed as fold-change over the sham group. **f** Illustrative confocal images demonstrating efficient CPT1B expression in cardiomyocytes after systemic injection of AAV9-CPT1B; scale bar = 100 µm. **g** Statistical quantification of CPT1B protein level in the myocardium of mice subjected to the treatments shown. (n = 8, comparison sham group vs. AAV9-Luc-treated TAC-group: *p < 0.05, **p < 0.01, ***p < 0.001, comparison AAV9-treated TAC-group vs. AAV9-CPT1B treated TAC-group: #*p* < 0.05, ##*p* < 0.01, ###*p < *0.001, one-way ANOVA, quantitative data are presented as means ± SD)

To validate the in vitro results in vivo, we generated AAV serotype 9 vectors for cardiac delivery in mice. C57B6/N mice were injected intravenously via the tail vein with either AAV9-CPT1B (intervention group) or AAV9-Luc (control group) 2 weeks prior to transverse aortic constriction (Fig. 5a). TAC-operated mice progressively developed heart failure, as illustrated by the deterioration in ejection fraction and fractional shortening, and increased left ventricular end-diastolic diameters (Fig. 5b-d). AAV9-CPT1B treatment prevented the drop in ejection fraction and fractional shortening 1; left ventricular dilatation was also decreased, but not significantly compared to the AAV9-LUC group (Fig. 5b, c, d, Supplementary Table). As expected, qPCR and immunohistochemical analyses revealed a markedly lower level of CPT1B mRNA and protein after TAC surgery and a significant overexpression of CPT1B in AAV9-CPT1B-treated mice on both mRNA (Fig. 5e) and protein levels (Fig. 5f, g).

**Fig. 6:**
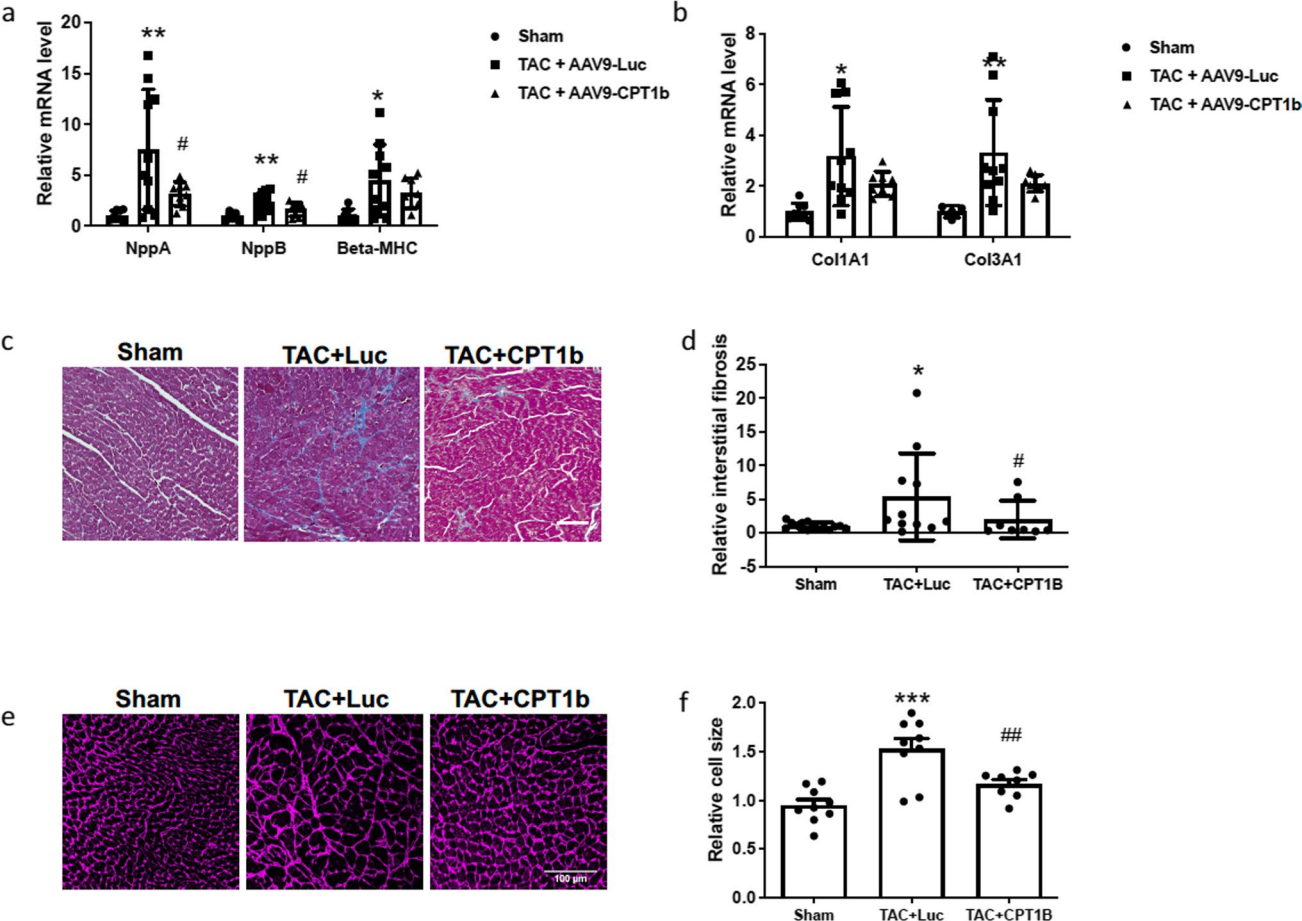
AAV9-mediated overexpression of CPT1B attenuates cardiac hypertrophy and extracellular matrix deposition. **a** Quantification of *Nppa*, *Nppb*, and *Myh7* mRNA levels as markers of a pro-hypertrophic response in mice subjected to the treatments shown. Values were normalized to GAPDH, which served as housekeeping gene and expressed as fold-change over the sham group. **b** Quantification of *Col1a1* and *Col3a1* mRNA levels as markers of cardiac fibrosis in mice subjected to the treatments shown. Values were normalized to GAPDH, which served as housekeeping gene and expressed as fold-change over the sham group. **c** Illustrative images showing heart sections with Masson’s trichrome staining to illustrate pathological extracellular matrix deposition of groups as indicated (scale bar = 100 µm) and **d** statistical quantification of the percentage of blue area marking pathological extracellular matrix deposition (40 images analyzed per group). Values were expressed as fold-changed over the sham group. **e** Illustrative images of heart sections stained with WGA as a membrane marker (red) on which cell size measurements shown in Fig. 3f were performed; scale bar = 100 µm. **f** Statistical quantification of relative cell area of WGA-stained heart sections in the designated treatment groups. Values were expressed as fold-changed over the sham group. (*n* = 8, comparison sham group vs. AAV9-Luc treated TAC-group: *p < 0.05, **p < 0.01, ***p < 0.001, comparison AAV9-treated TAC-group vs. AAV9-CPT1B-treated TAC-group: #*p* < 0.05, ##*p* < 0.01, ###*p < *0.001, quantitative data are presented as means ± SD)

In addition, pressure overload-induced upregulation of the cardiac pro-hypertrophic gene program, which was significantly prevented by CPT1B overexpression (Fig. 6a). Accordingly, cell size measurements showed pressure overload-induced cardiac hypertrophy, which was prevented by AAV9-mediated CPT1B overexpression (Fig. 6b, c). In addition, TAC-induced pressure overload resulted in increased extracellular matrix deposition, as demonstrated by Masson’s trichrome staining and upregulation of the fibrosis genes collagen 1 and collagen 3. The development of maladaptive fibrosis could be partially alleviated by CPT1B overexpression (Fig. 6d, e, f), while the effect on ventricular mass was not significantly different (Suppl. Table 1). Thus, the effectiveness of the CPT1B-expressing vector for the treatment of heart failure could also be demonstrated in vivo.

## Discussion

In this study, we show that cardiac-specific overexpression of CPT1B results in improved cardiac function and decreased cardiac hypertrophy and pathologic remodeling in a mouse model of pressure-overload-induced myocardial hypertrophy.

Heart failure is associated with a profoundly altered metabolism that relies primarily on glucose oxidation rather than fatty acid oxidation [11, 21]. Consistently, patients with DCM showed a significant reduction in fatty acid oxidation compared to healthy controls [8, 33]. On the other hand, obesity is associated with an increase in fatty acid uptake and oxidation, and circulating fatty acids have immense detrimental effects on cardiac contractility in obesity [24], underscoring that cardiac metabolism cannot be assessed independently of the underlying disease.

Increasing the level of fatty acid oxidation is expected to cause the heart muscle to rely less on glucose metabolism for ATP generation. CPT1B plays an essential role in fatty acid oxidation and is the predominantly expressed isoform of CPT1 in the heart. Therefore, CPT1B may be a suitable target to increase cardiac fatty acid oxidation. An interesting finding of our study supporting this approach is the marked reduction in CPT1B mRNA levels in cardiac tissue of patients with heart failure of different etiologies, namely HCM, DCM, and ICM. This observation supports previous studies [37]. However, the role of CPT1B in skeletal muscle and the heart under various stress conditions has not been finally resolved.

In the present study, we used AAV vectors to overexpress CPT1B in a murine pressure overload model induced by TAC. Under pressure overload conditions, CPT1B overexpression improved left ventricular function and cardiomyocyte hypertrophy, downregulated genes of the fetal gene program, and partially attenuated fibrosis. In agreement with our study, heterozygous CPT1B knock-out mice led to enhanced development of heart failure under pathological stress induced by TAC. The heterozygous mice showed apoptosis of cardiomyocytes, impaired mitochondrial bioenergetics, and accumulation of myocardial lipids [14]. Consistent with these observations, heart- and skeletal muscle-specific deletion of CPT1B in mice resulted in cardiac hypertrophy without induced stress and premature mortality at 15 weeks of age [13]. Similarly, inhibition of CPT1B by administration of 2-tetradecylglydicic acid in rats resulted in cardiac hypertrophy due to activation of angiotensin II, type 1 receptor signaling [51]. Consistent with our study, adenovirus-mediated overexpression of CPT1B led to an improvement in lipid accumulation in prohibitin-2-deficient neonatal cardiomyocytes in vitro. Interestingly, overexpression of CPT1B partially restored the oxygen consumption rate and fatty acid oxidation to baseline values [52]. Furthermore, global CPT1B knock-out animals showed early embryonic mortality [18]. As AAV-mediated overexpression of CPT1B in NRVCM increased the level of fatty acid oxidation and decreased the glycolytic capacity of NRVCMs in our study, normalization of dysregulated metabolic pathways might contribute to the therapeutic effect. 

In sharp contrast, blocking fatty acid oxidation via inhibition of CPT1 has been tested as a therapeutic approach in different models of heart failure. In aortic constriction-induced heart failure, chronic treatment with the CPT1 inhibitor etomoxir improved LV function and slowed the transition from compensated to failing hypertrophy in rats [46]. Similarly, treatment of dogs with pacing-induced heart failure using the CPT1 inhibitor oxfenicine prevented ventricular remodeling, attenuated LV dilation, and prolonged the time to reach end-stage heart failure [23]. In addition, etomoxir improved LV function in a small cohort of patients with heart failure (ischemic or idiopathic cardiomyopathy) [42]. However, the administration of etomoxir failed to reverse already-established myocardial hypertrophy in mice [43]. Moreover, CPT1B inhibition enhanced cardiac hypertrophy in other animal models [41, 54]. It also acutely decreased serum-free fatty acids in patients with heart failure, which led to a deterioration of myocardial efficiency, indicating a dependency on free fatty acid availability of the failing heart [47]. The different effects of pharmacological CPT1 inhibitors may be due to the extent of CPT1B downregulation or the pathophysiology of the particular diseases studied. In addition, some beneficial or detrimental effects may be partly due to unspecific side effects [54]. Taken together, a reduced CPT1B activity may play a detrimental role in the development of cardiac hypertrophy, and our data suggest a protective role of CPT1B overexpression. 

Other mechanistic studies supporting our hypothesis underlined that etomoxir treatment induces cardiac hypertrophy via NF-κB activation and increased oxidative stress [5]. In addition, inhibition of CPT1B in rats led to cardiomyocyte stress by activation of angiotensin II type 1 receptor [50]. These signaling pathways could therefore be responsible for the protective effect of AAV-mediated CPT1B overexpression in our experimental setup.

An important observation of our study is decreased oxidative stress following CPT1B overexpression in NRVCMs exposed to pro-hypertrophic stimuli. Indeed, oxidative stress was demonstrated to be a hallmark of heart failure and correlates with the severity of cardiac dysfunction in patients [3]. Overactive oxygen species directly contribute to pathophysiological events such as arrhythmias, cardiomyocyte apoptosis, abnormal calcium signaling, and mitochondrial damage [32]. Importantly, changes in mitochondrial homeostasis, integrity, and recycling have been demonstrated to be associated with decreased ATP production, decreased contractility, and, ultimately, heart failure [22]. Interestingly, CPT1C overexpression in neurons alleviated oxidative stress and blunted apoptosis in neurons in a model of Alzheimer’s disease [9]. Similarly, stress conditions that lead to increased Ca^2+^ overload and ROS generation induce loss of mitochondrial potential in cardiac myocytes due to the opening of the mitochondrial permeability transition pore, further triggering apoptosis, impaired contractility, and pro-inflammatory responses [55]. Here, we provide evidence that in vitro CPT1B overexpression can rescue mitochondrial function by preserving its membrane potential. This observation likely explains the significant beneficial effects noted in TAC-treated animals receiving gene therapy.

The question of whether FAO activation could be beneficial in heart failure is complex and depends on several factors, including the stage of disease and the metabolic state of the myocardium. On one hand, studies have demonstrated that reducing FAO leads to exacerbated cardiac dysfunction in several mouse models [1, 14, 44]. This suggests that, in some contexts, enhancing FAO could reverse the metabolic inefficiency characteristic of HF, when myocardial energy production is severely impaired. In line with these findings, recent studies in hiPSC-derived cardiomyocytes have provided evidence that enhancing FAO improves mitochondrial function and contractility, even in human-derived cells [53]. These findings are particularly important as they provide insight into the potential therapeutic applications of FAO modulation in human heart failure. Importantly, our study provides additional evidence supporting this hypothesis, demonstrating the beneficial effects of CPT1B overexpression in a human heart failure model using hiPSC-CM. However, the benefits of FAO activation are not entirely understood. Excessive reliance on FAO can lead to the accumulation of lipid intermediates [27], promoting mitochondrial dysfunction, oxidative stress, and even exacerbate myocardial injury. Therefore, while FAO modulation holds potential as a therapeutic strategy in heart failure, its efficacy and safety in heart failure treatment depend on maintaining a balance. Future studies should focus on understanding the specific conditions under which FAO activation is beneficial, particularly in human models, and how these findings can be translated into clinical therapies.

Our study is subject to several limitations that could be addressed in future studies. Primarily, the experimental design focuses on a preventive approach involving the administration of AAV9 prior to TAC. For further translation, a therapeutic strategy in which mice are injected after surgery should be considered. Second, although unlikely, an effect on blood pressure cannot be finally ruled out. Third, although TAC is a widely used model for inducing cardiac hypertrophy, other relevant models have been described in various studies, for example, angiotensin II or isoproterenol infusion-based models [38]. Importantly, angiotensin II has been shown to downregulate the fatty acid oxidation pathway in cardiomyocytes, which could be compensated by CPT1B overexpression, as proposed in our study [36]. Likewise, isoprenaline markedly affected fatty acid oxidation in HL-1 cells, leading to cardiomyocyte apoptosis [36]. Hence, we hypothesize that CPT1B overexpression might be protective in these hypertrophy models. In addition, we chose neonatal cardiomyocytes as one of our in vitro system to test our hypotheses. Although these cells present with a low degree of fatty acid oxidation as compared to adult cardiomyocytes [28], they retain their phenotype longer in cell culture conditions and survive longer in vitro, allowing efficient transduction with AAVs [2]. Moreover, it was demonstrated that fatty acid utilization in NRVCMs is significantly decreased upon PE treatment, leading to cardiomyocyte hypertrophy through NF-κB activation [2].

Taken together, we found that overexpression of CPT1B results in attenuated cardiomyocyte hypertrophy and reduced mitochondrial reactive oxygen species in vitro associated with normalization of dysregulated metabolic pathways. AAV-mediated gene transfer of CPT1B in mice prevented the development of TAC-induced left ventricular systolic dysfunction and cardiac fibrosis, suggesting that upregulation of CPT1B expression could be a promising approach for heart failure treatment.

## Supplementary Information

Below is the link to the electronic supplementary material.

Supplementary file1 (DOCX 142 KB)

## Data Availability

Any materials can be obtained from the authors upon request.

## References

[1] Augustus AS, Buchanan J, Park TS, Hirata K, Noh HL, Sun J, Homma S, D’Armiento J, Abel ED, Goldberg IJ (2006) Loss of lipoprotein lipase-derived fatty acids leads to increased cardiac glucose metabolism and heart dysfunction. J Biol Chem 281:8716–8723. 10.1074/jbc.M509890200 16410253 10.1074/jbc.M509890200

[2] Banyasz T, Lozinskiy I, Payne CE, Edelmann S, Norton B, Chen B, Chen-Izu Y, Izu LT, Balke CW (2008) Transformation of adult rat cardiac myocytes in primary culture. Exp Physiol 93(3):370–382. 10.1113/expphysiol.2007.040659 18156167 10.1113/expphysiol.2007.040659

[3] Belch JJF, Bridges AB, Scott N, Chopra M (1991) Oxygen free radicals and congestive heart failure. Heart 65(5):245–248. 10.1136/hrt.65.5.245 10.1136/hrt.65.5.245 PMC1024624 2039668

[4] Blair CA, Brundage EA, Thompson KL, Stromberg A, Guglin M, Biesiadecki BJ, Campbell KS (2020) Heart failure in humans reduces contractile force in myocardium from both ventricles. JACC Basic Transl Sci 5:786–798. 10.1016/j.jacbts.2020.05.014 32875169 10.1016/j.jacbts.2020.05.014 PMC7452203

[5] Cabrero A, Merlos M, Laguna JC, Carrera MV (2003) Down-regulation of acyl-CoA oxidase gene expression and increased NF-κB activity in etomoxir-induced cardiac hypertrophy. J Lipid Res 44:388–398. 10.1194/jlr.M200294-JLR200 12576521 10.1194/jlr.M200294-JLR200

[6] Chen J, Yue F, Kuang S (2022) Labeling and analyzing lipid droplets in mouse muscle stem cells. STAR Protoc 3:101849. 10.1016/j.xpro.2022.101849 36595920 10.1016/j.xpro.2022.101849 PMC9679676

[7] Chen S, Zou Y, Song C, Cao K, Cai K, Wu Y, Zhang Z, Geng D, Sun W, Ouyang N, Zhang N, Li Z, Sun G, Zhang Y, Sun Y, Zhang Y (2023) The role of glycolytic metabolic pathways in cardiovascular disease and potential therapeutic approaches. Basic Res Cardiol 118:48. 10.1007/s00395-023-01018-w 37938421 10.1007/s00395-023-01018-w PMC10632287

[8] Davila-Roman VG, Vedala G, Herrero P, de las Fuentes L, Rogers JG, Kelly DP, Gropler RJ (2002) Altered myocardial fatty acid and glucose metabolism in idiopathic dilated cardiomyopathy. J Am Coll Cardiol 40:271–277. 10.1016/s0735-1097(02)01967-8 12106931 10.1016/s0735-1097(02)01967-8

[9] Ding Y, Zhang H, Liu Z, Li Q, Guo Y, Chen Y, Chang Y, Cui H (2021) Carnitine palmitoyltransferase 1 (CPT1) alleviates oxidative stress and apoptosis of hippocampal neuron in response to beta-Amyloid peptide fragment Abeta(25–35). Bioengineered 12:5440–5449. 10.1080/21655979.2021.1967032 34424821 10.1080/21655979.2021.1967032 PMC8806834

[10] Doenst T, Nguyen TD, Abel ED (2013) Cardiac metabolism in heart failure: Implications beyond atp production. Circ Res 113:709–724. 10.1161/circresaha.113.300376 23989714 10.1161/CIRCRESAHA.113.300376 PMC3896379

[11] Fillmore N, Mori J, Lopaschuk GD (2014) Mitochondrial fatty acid oxidation alterations in heart failure, ischaemic heart disease and diabetic cardiomyopathy. In:Br J Pharmacol, p 2080–2090 10.1111/bph.12475 PMC3976623 24147975

[12] Giovou AE, Gladka MM, Christoffels VM (2024) The impact of natriuretic peptides on heart development, homeostasis, and disease. Cells. 10.3390/cells13110931 38891063 10.3390/cells13110931 PMC11172276

[13] Haynie KR, Vandanmagsar B, Wicks SE, Zhang J, Mynatt RL (2014) Inhibition of carnitine palymitoyltransferase1b induces cardiac hypertrophy and mortality in mice. Diabetes Obes Metab 16:757–760. 10.1111/dom.12248 24330405 10.1111/dom.12248 PMC4057362

[14] He L, Kim T, Long Q, Liu J, Wang P, Zhou Y, Ding Y, Prasain J, Wood PA, Yang Q (2012) Carnitine palmitoyltransferase-1b deficiency aggravates pressure overload-induced cardiac hypertrophy caused by lipotoxicity. Circulation 126:1705–1716. 10.1161/circulationaha.111.075978 22932257 10.1161/CIRCULATIONAHA.111.075978 PMC3484985

[15] Hernandez-Resendiz S, Prakash A, Loo SJ, Semenzato M, Chinda K, Crespo-Avilan GE, Dam LC, Lu S, Scorrano L, Hausenloy DJ (2023) Targeting mitochondrial shape: at the heart of cardioprotection. Basic Res Cardiol 118:49. 10.1007/s00395-023-01019-9 37955687 10.1007/s00395-023-01019-9 PMC10643419

[16] Heusch G (2022) Coronary blood flow in heart failure: cause, consequence and bystander. Basic Res Cardiol 117:1. 10.1007/s00395-022-00909-8 35024969 10.1007/s00395-022-00909-8 PMC8758654

[17] Heusch G, Andreadou I, Bell R, Bertero E, Botker HE, Davidson SM, Downey J, Eaton P, Ferdinandy P, Gersh BJ, Giacca M, Hausenloy DJ, Ibanez B, Krieg T, Maack C, Schulz R, Sellke F, Shah AM, Thiele H, Yellon DM, Di Lisa F (2023) Health position paper and redox perspectives on reactive oxygen species as signals and targets of cardioprotection. Redox Biol 67:102894. 10.1016/j.redox.2023.102894 37839355 10.1016/j.redox.2023.102894 PMC10590874

[18] Ji S, You Y, Kerner J, Hoppel CL, Schoeb TR, Chick WSH, Hamm DA, Sharer JD, Wood PA (2008) Homozygous carnitine palmitoyltransferase 1b (muscle isoform) deficiency is lethal in the mouse. Mol Genet Metab 93(3):314–322. 10.1016/j.ymgme.2007.10.006 18023382 10.1016/j.ymgme.2007.10.006 PMC2270477

[19] Karwi QG, Uddin GM, Ho KL, Lopaschuk GD (2018) Loss of metabolic flexibility in the failing heart. Front Cardiovasc Med. 10.3389/fcvm.2018.00068 29928647 10.3389/fcvm.2018.00068 PMC5997788

[20] Lee L, Campbell R, Scheuermann-Freestone M, Taylor R, Gunaruwan P, Williams L, Ashrafian H, Horowitz J, Fraser AG, Clarke K, Frenneaux M (2005) Metabolic modulation with perhexiline in chronic heart failure: A randomized, controlled trial of short-term use of a novel treatment. Circulation 112:3280–3288. 10.1161/circulationaha.105.551457 16301359 10.1161/CIRCULATIONAHA.105.551457

[21] Lei B, Lionetti V, Young ME, Chandler MP, D’Agostino C, Kang E, Altarejos M, Matsuo K, Hintze TH, Stanley WC, Recchia FA (2004) Paradoxical downregulation of the glucose oxidation pathway despite enhanced flux in severe heart failure. J Mol Cell Cardiol 36:567–576. 10.1016/j.yjmcc.2004.02.004 15081316 10.1016/j.yjmcc.2004.02.004

[22] Lesnefsky EJ, Moghaddas S, Tandler B, Kerner J, Hoppel CL (2001) Mitochondrial dysfunction in cardiac disease: Ischemia - reperfusion, aging, and heart failure. J Mol Cell Cardiol 33:1065–1089. 10.1006/jmcc.2001.1378 11444914 10.1006/jmcc.2001.1378

[23] Lionetti V, Linke A, Chandler MP, Young ME, Penn MS, Gupte S, D’Agostino C, Hintze TH, Stanley WC, Recchia FA (2005) Carnitine palmitoyl transferase-I inhibition prevents ventricular remodeling and delays decompensation in pacing-induced heart failure. Cardiovasc Res 66:454–461. 10.1016/j.cardiores.2005.02.004 15914110 10.1016/j.cardiores.2005.02.004

[24] Lopaschuk GD, Folmes CDL, Stanley WC (2007) Cardiac energy metabolism in obesity. Circ Res 101:335–347. 10.1161/circresaha.107.150417 17702980 10.1161/CIRCRESAHA.107.150417

[25] Lopaschuk GD, Karwi QG, Tian R, Wende AR, Abel ED (2021) Cardiac energy metabolism in heart failure. Circ Res 128:1487–1513. 10.1161/circresaha.121.318241 33983836 10.1161/CIRCRESAHA.121.318241 PMC8136750

[26] Lopaschuk GD, Ussher JR, Folmes CDL, Jaswal JS, Stanley WC (2010) Myocardial fatty acid metabolism in health and disease. Physiol Rev. 10.1152/physrev.00015.2009 20086077 10.1152/physrev.00015.2009

[27] Ly LD, Xu S, Choi SK, Ha CM, Thoudam T, Cha SK, Wiederkehr A, Wollheim CB, Lee IK, Park KS (2017) Oxidative stress and calcium dysregulation by palmitate in type 2 diabetes. Exp Mol Med 49:e291. 10.1038/emm.2016.157 28154371 10.1038/emm.2016.157 PMC5336562

[28] Maroli G, Braun T (2021) The long and winding road of cardiomyocyte maturation. Cardiovasc Res 117:712–726. 10.1093/cvr/cvaa159 32514522 10.1093/cvr/cvaa159

[29] Mosqueira D, Mannhardt I, Bhagwan JR, Lis-Slimak K, Katili P, Scott E, Hassan M, Prondzynski M, Harmer SC, Tinker A, Smith JGW, Carrier L, Williams PM, Gaffney D, Eschenhagen T, Hansen A, Denning C (2018) CRISPR/Cas9 editing in human pluripotent stem cell-cardiomyocytes highlights arrhythmias, hypocontractility, and energy depletion as potential therapeutic targets for hypertrophic cardiomyopathy. Eur Heart J 39:3879–3892. 10.1093/eurheartj/ehy249 29741611 10.1093/eurheartj/ehy249 PMC6234851

[30] Muller OJ, Heckmann MB, Ding L, Rapti K, Rangrez AY, Gerken T, Christiansen N, Rennefahrt UEE, Witt H, Gonzalez Maldonado S, Ternes P, Schwab DM, Ruf T, Hille S, Remes A, Jungmann A, Weis TM, Kreusser JS, Grone HJ, Backs J, Schatz P, Katus HA, Frey N (2019) Comprehensive plasma and tissue profiling reveals systemic metabolic alterations in cardiac hypertrophy and failure. Cardiovasc Res 115:1296–1305. 10.1093/cvr/cvy274 30418544 10.1093/cvr/cvy274

[31] Muller OJ, Leuchs B, Pleger ST, Grimm D, Franz WM, Katus HA, Kleinschmidt JA (2006) Improved cardiac gene transfer by transcriptional and transductional targeting of adeno-associated viral vectors. Cardiovasc Res 70:70–78. 10.1016/j.cardiores.2005.12.017 16448634 10.1016/j.cardiores.2005.12.017

[32] Munzel T, Camici GG, Maack C, Bonetti NR, Fuster V, Kovacic JC (2017) Impact of oxidative stress on the heart and vasculature: part 2 of a 3-part series. J Am Coll Cardiol 70:212–229. 10.1016/j.jacc.2017.05.035 28683969 10.1016/j.jacc.2017.05.035 PMC5663297

[33] Neglia D, Caterina AD, Marraccini P, Natali A, Ciardetti M, Vecoli C, Gastaldelli A, Ciociaro D, Pellegrini P, Testa R, Menichetti L, L’Abbate A, Stanley WC, Recchia FA (2007) Impaired myocardial metabolic reserve and substrate selection flexibility during stress in patients with idiopathic dilated cardiomyopathy. American Journal of Physiology - Heart and Circulatory Physiology 293:3270–3278. 10.1152/ajpheart.00887.2007 10.1152/ajpheart.00887.2007 17921325

[34] Neubauer S (2007) The Failing Heart — An Engine Out of Fuel. N Engl J Med 356:1140–1151. 10.1056/nejmra063052 17360992 10.1056/NEJMra063052

[35] Osorio JC, Stanley WC, Linke A, Castellari M, Diep QN, Panchal AR, Hintze TH, Lopaschuk GD, Recchia FA (2002) Circulation 106:606–612. 10.1161/01.Cir.0000023531.22727.C1 12147544 10.1161/01.cir.0000023531.22727.c1

[36] Pellieux C, Montessuit C, Papageorgiou I, Lerch R (2009) Angiotensin II downregulates the fatty acid oxidation pathway in adult rat cardiomyocytes via release of tumour necrosis factor-α. Cardiovasc Res 82:341–350. 10.1093/cvr/cvp004 19131364 10.1093/cvr/cvp004

[37] Razeghi P, Young ME, Alcorn JL, Moravec CS, Frazier OH, Taegtmeyer H (2001) Metabolic gene expression in fetal and failing human heart. Circulation 104:2923–2931. 10.1161/hc4901.100526 11739307 10.1161/hc4901.100526

[38] Riehle C, Bauersachs J (2019) Small animal models of heart failure. Cardiovasc Res 115:1838–1849. 10.1093/cvr/cvz161 31243437 10.1093/cvr/cvz161 PMC6803815

[39] Ritterhoff J, Tian R (2017) Metabolism in cardiomyopathy: every substrate matters. Cardiovasc Res. 10.1093/cvr/cvx017 28395011 10.1093/cvr/cvx017 PMC5852620

[40] Rockman HA, Ross RS, Harris AN, Knowlton KU, Steinhelper ME, Field LJ, Ross J, Chien KR (1991) Segregation of atrial-specific and inducible expression of an atrial natriuretic factor transgene in an in vivo murine model of cardiac hypertrophy. Proc Natl Acad Sci USA 88:8277–8281. 10.1073/pnas.88.18.8277 1832775 10.1073/pnas.88.18.8277 PMC52490

[41] Rupp H, Elimban V, Dhalla NS (1992) Modification of subcellular organelles in pressure-overloaded heart by etomoxir, a carnitine palmitoyltransferase I inhibitor. FASEB J 6:2349–2353. 10.1096/fasebj.6.6.1531968 1531968 10.1096/fasebj.6.6.1531968

[42] Schmidt-Schweda S, Holubarsch C (2000) First clinical trial with etomoxir in patients with chronic congestive heart failure. Clin Sci (Lond) 99:27–35 10887055

[43] Schwarzer M, Faerber G, Rueckauer T, Blum D, Pytel G, Mohr FW, Doenst T (2009) The metabolic modulators, Etomoxir and NVP-LAB121, fail to reverse pressure overload induced heart failure in vivo. Basic Res Cardiol 104:547–557. 10.1007/s00395-009-0015-5 19294446 10.1007/s00395-009-0015-5

[44] Steinbusch LK, Luiken JJ, Vlasblom R, Chabowski A, Hoebers NT, Coumans WA, Vroegrijk IO, Voshol PJ, Ouwens DM, Glatz JF, Diamant M (2011) Absence of fatty acid transporter CD36 protects against Western-type diet-related cardiac dysfunction following pressure overload in mice. Am J Physiol Endocrinol Metab 301:E618-627. 10.1152/ajpendo.00106.2011 21712535 10.1152/ajpendo.00106.2011

[45] Turcani M, Rupp H (1997) Etomoxir improves left ventricular performance of pressure-overloaded rat heart. Circulation 96:3681–3686. 10.1161/01.Cir.96.10.3681 9396471 10.1161/01.cir.96.10.3681

[46] Turcani M, Rupp H (1999) Modification of left ventricular hypertrophy by chronic etomoxir treatment. Br J Pharmacol 126:501–507. 10.1038/sj.bjp.0702312 10077244 10.1038/sj.bjp.0702312 PMC1565820

[47] Tuunanen H, Engblom E, Naum A, Någren K, Hesse B, Airaksinen KEJ, Nuutila P, Iozzo P, Ukkonen H, Opie LH, Knuuti J (2006) Free fatty acid depletion acutely decreases cardiac work and efficiency in cardiomyopathic heart failure. Circulation 114:2130–2137. 10.1161/circulationaha.106.645184 17088453 10.1161/CIRCULATIONAHA.106.645184

[48] Weis BC, Cowan AT, Brown N, Foster DW, McGarry JD (1994) Use of a selective inhibitor of liver carnitine palmitoyltransferase I (CPT I) allows quantification of its contribution to total CPT I activity in rat heart. Evidence that the dominant cardiac CPT I isoform is identical to the skeletal muscle enzyme. J Biol Chem 269:26443–26448. 10.1016/s0021-9258(18)47214-6 7929365

[49] Werfel S, Jungmann A, Lehmann L, Ksienzyk J, Bekeredjian R, Kaya Z, Leuchs B, Nordheim A, Backs J, Engelhardt S, Katus HA, Müller OJ (2014) Rapid and highly efficient inducible cardiac gene knockout in adult mice using AAV-mediated expression of Cre recombinase. Cardiovasc Res 104:15–23. 10.1093/cvr/cvu174 25082846 10.1093/cvr/cvu174

[50] Wolkowicz PE, Urthaler F, Forrest C, Shen H, Durand J, Wei CC, Oparil S, Dell’Italia LJ (1999) 2-Tetradecylglycidic acid, an inhibitor of carnitine palmitoyltransferase-1, induces myocardial hypertrophy via the AT1 receptor. J Mol Cell Cardiol 31:1405–1412. 10.1006/jmcc.1999.0977 10424880 10.1006/jmcc.1999.0977

[51] Wolkowicz PE, Urthaler F, Forrest C, Shen H, Durand J, Wei CC, Oparil S, Dell’Italia LJ (1999) 2-Tetradecylglycidic acid, an inhibitor of carnitine palmitoyltransferase-1, induces myocardial hypertrophy via the AT1 receptor. J Mol Cell Cardiol 31:1405–1412. 10.1006/jmcc.1999.0977 10424880 10.1006/jmcc.1999.0977

[52] Wu D, Jian C, Peng Q, Hou T, Wu K, Shang B, Zhao M, Wang Y, Zheng W, Ma Q, Li CY, Cheng H, Wang X, Zhao L (2020) Prohibitin 2 deficiency impairs cardiac fatty acid oxidation and causes heart failure. Cell Death Dis 11:181–181. 10.1038/s41419-020-2374-7 32165613 10.1038/s41419-020-2374-7 PMC7067801

[53] Yang X, Rodriguez ML, Leonard A, Sun L, Fischer KA, Wang Y, Ritterhoff J, Zhao L, Kolwicz SC Jr, Pabon L, Reinecke H, Sniadecki NJ, Tian R, Ruohola-Baker H, Xu H, Murry CE (2019) Fatty Acids Enhance the Maturation of Cardiomyocytes Derived from Human Pluripotent Stem Cells. Stem Cell Reports 13:657–668. 10.1016/j.stemcr.2019.08.013 31564645 10.1016/j.stemcr.2019.08.013 PMC6829750

[54] Yotsumoto T, Naitoh T, Kitahara M, Tsuruzoe N (2000) Effects of carnitine palmitoyltransferase I inhibitors on hepatic hypertrophy. Eur J Pharmacol 398:297–302. 10.1016/s0014-2999(00)00288-0 10854842 10.1016/s0014-2999(00)00288-0

[55] Zhou B, Tian R (2018) Mitochondrial dysfunction in pathophysiology of heart failure. J Clin Invest. 10.1172/JCI120849 30124471 10.1172/JCI120849 PMC6118589

