# Supplementary material for: AAV-mediated overexpression of CPT1B protects from cardiac hypertrophy and heart failure in a murine pressure overload model
Source: Basic Res Cardiol. 2025 Jul 11;120(5):975–89. doi: 10.1007/s00395-025-01123-y (PMC12518452; doi:10.1007/s00395-025-01123-y)
Supplement: Supplementary file 1 — Supplementary file1 (DOCX 142 KB) [file 395_2025_1123_MOESM1_ESM.docx]

Supplementary material

**AAV-mediated overexpression of CPT1B protects from cardiac hypertrophy and heart failure in a murine pressure overload model**

Anca Remes^,1,2†^, Theresa Ruf^3,4†^, Tinatin Zurashvili^1,5^, Lin Ding^3,6^, Moritz Meyer-Jens^2,7^, Dominic M. Schwab^3^, Susanne Hille^1,2^, Andrea Matzen^2,6^, Sabine Michalewski^1,2^, Lucia Kilian^2,6^, Prithviraj Manohar Vijaya Shetty^1,2^, Marie-Christin Fuchs^3^, Matthias Eden^3,4,6^, Hermann-Josef Gröne^8^, Kleopatra Rapti^3^, Andreas Jungmann^3^, Hendrik Milting^9^, Hugo A. Katus^3,4^, Lucie Carrier^2,7^, Derk Frank^2,6^, Norbert Frey^3,4^, Oliver J. Müller^1,2^

^1^ Department of Internal Medicine V, University of Kiel, Kiel, Germany

^2^ German Centre for Cardiovascular Research, Partner Site Hamburg/Kiel/Lübeck, Kiel, Germany

^3^ Internal Medicine III, University Hospital Heidelberg, Heidelberg, Germany

^4^ German Centre for Cardiovascular Research, Partner Site Heidelberg/Mannheim, Heidelberg, Germany

^5^ David Tvildiani Medical University, Tbilisi, Georgia

^6^ Department of Internal Medicine III, University of Kiel, Kiel, Germany

^7^ Department of Experimental Pharmacology and Toxicology, University Medical Center Hamburg Eppendorf, Hamburg, Germany

^8^ Department for Cellular and Molecular Pathology, DKFZ Heidelberg, Germany

^9^ Erich & Hanna Klessmann-Institut, Ruhr-Universität Bochum, Herz & Diabeteszentrum NRW, Bad Oeynhausen, Germany

^†^ These authors contributed equally to this work.

Corresponding author: Oliver J. Müller, Department of Internal Medicine V, University of Kiel and University Hospital Schleswig-Holstein, Arnold-Heller-Str. 3, 24105 Kiel, Germany. Tel. +49 431 50022950; Fax +49 431 50023204; e-mail: [oliver.mueller@uksh.de](mailto:oliver.mueller@uksh.de)

ORCHID ID 0000-0001-8223-263

**
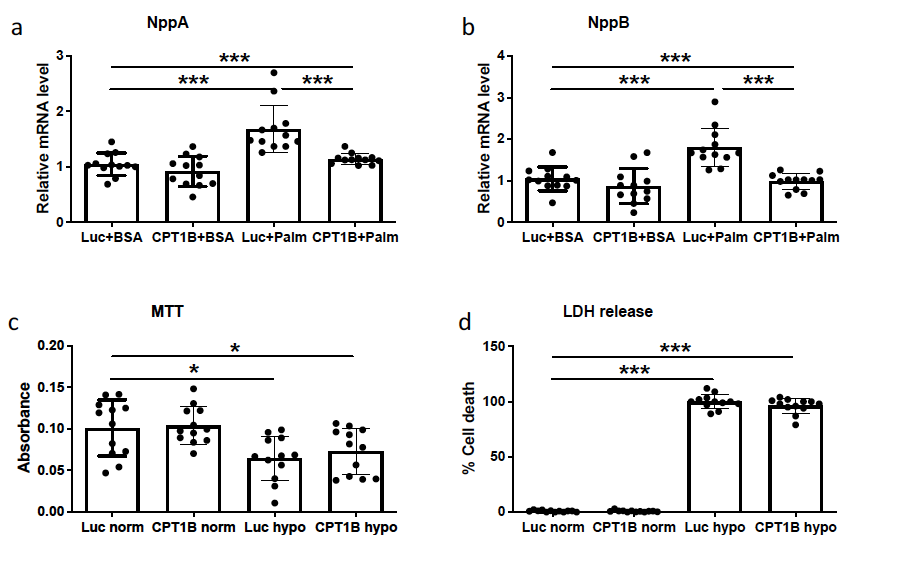
**

**Supplementary Fig. 1. CPT1B overexpression decreases palmitate-induced cardiomyocyte hypertrophy and does not affect hypoxia-induced dysfunction.** Statistical quantification of mRN levels of NppA **(a)** and NppB **(b)** as markers of pro-hypertrophic response in NRVCMs**.** RPL32 served as a housekeeping gene and values were normalized to Luc-BSA treated cells. **(c)** Assessment of total metabolic rate of NRVCMs in the depicted treatment groups, measured by MTT assay. **(d)** Quantification of LDH release in the supernatant in NRVCMs subjected to hypoxia. AAV6-Luc treated cells in hypoxia conditions were considered as control (100% LDH release). (*p<0.05, ***p<0.001, quantitative data are presented as means±SD, n=3).


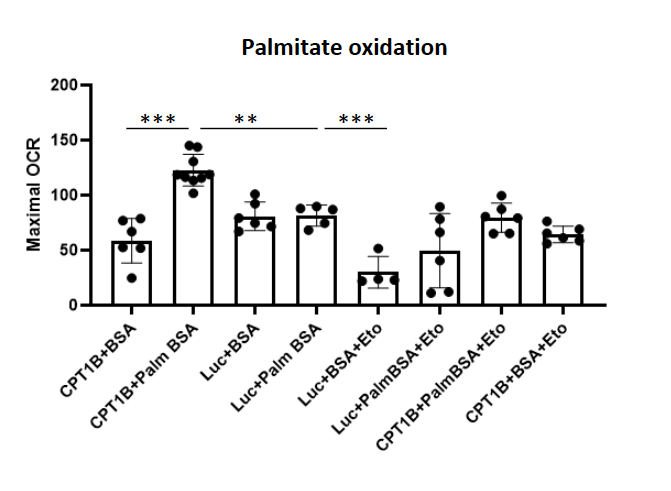


**Supplementary Fig. 2. CPT1B overexpression leads to increased palmitate oxidation in hiPSC-CM.** Shown is maximal oxygen consumption rate (OCR) in the depicted treatment groups, 3 days after AAV transduction. (**p<0.01, ***p<0.001, quantitative data are presented as means±SD, n=1 biological replicate and 6 technical replicates)

**Suppl. Table 1. Echocardiographic measurements of mice subjected to TAC**

| **Heart function prior to TAC (Baseline)** | | | | | |
| --- | --- | --- | --- | --- | --- |
|  | Sham  (n=14) | AAV9-LUC  (n=12) | AAV9-CPT1B  (n=8) | *ANOVA-Multiple-Comparison* | |
| Echo-Parameter | Mean ± SV | Mean ± SV | Mean ± SV | LUC vs. CPT1B | LUC vs. Sham |
| EF (%) | 53,79 ± 7,43 | 56,17 ± 7,42 | 54,2 ± 7,2 | ns | ns |
| FS (%) | 15,83 ± 2,82 | 21,4 ±2,48 | 21,87 ± 6,3 | ns | ns |
| LV Mass (mg) | 134,4 ± 6,65 | 117,9 ± 5,14 | 117,7 ± 4,12 | ns | ns |
| LVPW;d (mm) | 0,72 ± 0,029 | 0,70 ± 0,032 | 0,69 ± 0,039 | ns | ns |
| LVDD (mm) | 4,34 ± 0,095 | 4,39 ± 0,056 | 4,25 ± 0,12 | ns | ns |
| Heart rate | 447,15 ± 32,74 | 454,14 ± 48,91 | 423,15 ± 14,66 | ns | ns |
| **Heart function 2 weeks after TAC** | | | | | |
|  | Sham  (n=14) | AAV9-LUC  (n=12) | AAV9-CPT1B  (n=8) | *ANOVA-Multiple-Comparison* | |
| Echo-Parameter | Mean ± SV | Mean ± SV | Mean ± SV | LUC vs. CPT1B | LUC vs. Sham |
| EF (%) | 51,59 ± 9,75 | 42,82 ± 7,87 | 53,24 ± 9,29 | ns | ** |
| FS (%) | 26,53 ± 6,35 | 15,83 ± 2,82 | 23,44 ± 5,8 | ns | *** |
| LV Mass (mg) | 128,8 ± 5,54 | 164,6 ± 7,87 | 167,7 ± 8,92 | ns | *** |
| LVPW;d (mm) | 0,67 ± 0,018 | 0,87 ± 0,025 | 0,84 ± 0,045 | ns | *** |
| LVDD (mm) | 4,37 ± 0,070 | 4,54 ± 0,070 | 4,46 ± 0,060 | ns | ns |
| Heart rate | 484,88 ± 67,51 | 454,73 ± 27,62 | 484,26 ± 47,23 | ns | ns |
| **Heart function 4 weeks after TAC** | | | | | |
|  | Sham  (n=14) | AAV9-LUC  (n=12) | AAV9-CPT1B  (n=8) | *ANOVA-Multiple-Comparison* | |
| Echo-Parameter | Mean ± SV | Mean ± SV | Mean ± SV | LUC vs. CPT1B | LUC vs. Sham |
| EF (%) | 53,42 ± 7,99 | 34,42 ± 11,01 | 48,44 ± 17,95 | ** | *** |
| FS (%) | 23,93 ±5,19 | 23,93 ± 5,19 | 26,88 ± 8,1 | ** | *** |
| LV Mass (mg) | 124,7 ± 4,13 | 201,3 ± 20,01 | 189,0 ± 14,18 | ns | *** |
| LVPW;d (mm) | 0,70 ± 0,024 | 0,92 ± 0,051 | 1,01 ± 0,082 | ns | ** |
| LVDD (mm) | 4,34 ± 0,033 | 4,62 ± 0,11 | 4,23 ± 0,21 | ns | ns |
| Heart rate | 425,34 ± 33,01 | 428,13 ± 31,50 | 424,50 ± 35,31 | ns | ns |
| **Heart function 6 weeks after TAC** | | | | | |
|  | Sham  (n=14) | AAV9-LUC  (n=12) | AAV9-CPT1B  (n=8) | *ANOVA-Multiple-Comparison* | |
| Echo-Parameter | Mean ± SV | Mean ± SV | Mean ± SV | LUC vs. CPT1B | LUC vs. Sham |
| EF (%) | 47,40 ± 6,14 | 40,95 ± 11,85 | 51,55 ± 7,78 | * | *** |
| FS (%) | 25,4 ± 4,73 | 25,4 ± 4,73 | 20,59 ± 5,36 | * | *** |
| LV Mass (mg) | 126,1 ± 3,64 | 221,6 ± 14,14 | 207,2 ± 19,88 | ns | *** |
| LVPW;d (mm) | 0,65 ± 0,022 | 1,01 ± 0,048 | 0,98 ± 0,068 | ns | *** |
| LVDD (mm) | 4,31 ± 0,066 | 4,91 ± 0,15 | 4,61 ± 0,16 | ns | ** |
| Heart rate | 427,16 ± 24,7 | 421,31 ± 28,35 | 415,38 ± 21,53 | ns | ns |
